# Radiation-induced impairment of skeletal muscle regeneration

**DOI:** 10.2478/raon-2025-0048

**Published:** 2025-09-05

**Authors:** Maja Cemazar, Mihaela Jurdana

**Affiliations:** Faculty of Health Sciences, University of Primorska, Izola, Slovenia; Institute of Oncology Ljubljana, Ljubljana, Slovenia

**Keywords:** skeletal muscle, muscle microenvironment, radiotherapy, muscle regeneration, melatonine

## Abstract

**Background:**

Radiotherapy is a cornerstone of treatment for various cancers, but often causes collateral damage to surrounding healthy tissue, including skeletal muscle. Ionizing radiation leads to oxidative stress and inflammation, which impairs the regenerative capacity of muscle tissue. Irradiation reduces the number and functionality of satellite cells and disrupts the tightly regulated processes of myogenesis and tissue remodelling. In addition, irradiation alters the muscle microenvironment by promoting fibrosis and vascular damage, which further impedes effective regeneration. Cytokine signalling pathways are also dysregulated following irradiation, contributing to impaired activation and differentiation of satellite cells.

**Conclusions:**

There is evidence that factors such as melatonin and growth factors can improve muscle regeneration. Understanding the molecular and cellular mechanisms underlying the impairment of muscle regeneration after radiotherapy is crucial for the development of targeted strategies to mitigate side effects and improve patients’ quality of life. Overall, the preservation and restoration of muscle function in irradiated tissue remains a critical challenge that requires multidisciplinary approaches.

## Introduction

Radiotherapy remains one of the most important component of cancer treatment with approximately 50% of all cancer patients receiving radiation therapy during their course of illness; it accounts for 40% of cancer’s curative treatments.^1^ The main goal of radiation therapy is to deprive cancer cells of their proliferation (cell division) potential. X-rays, gamma rays and charged particles are the most types of radiation used for cancer treatment.

The biological effectiveness (cell killing) of radiation is influenced by factors such as linear energy transfer (LET), total dose, fractionation scheme, and the radio-sensitivity of the targeted cells or tissues.^2,3^ Low LET radiation transfers a smaller amount of energy, while high LET radiation delivers a higher dose of energy to the targeted areas. Although radiation is aimed at destroying tumour cells, it is unavoidable that surrounding healthy tissues may also suffer damage. The primary goal of radiation therapy is to deliver the highest possible dose to tumour cells while minimizing the exposure to normal, healthy tissues.^4^

Skeletal muscle is one of the most dynamic and plastic tissues of the human body. In humans, skeletal muscle comprises approximately 40% of total body weight and is often exposed to ionizing radiation during radiotherapeutic treatment. Many studies have explored the effects of radiation on skeletal muscle, demonstrating that muscle damage from irradiation can persist for many years.^5^

The effects of ionizing radiation on skeletal muscle can be categorized into early and late effects, each exhibiting distinct patterns of response to fractionations and dose-response relations. Early effects, characterized by acute damage, occur rapidly, in a few days after exposure, influencing or damaging muscle stem (satellite) cells.^5^ On the other hand, late effects appear after a delay of months or years and can lead to biological changes in skeletal muscle. The severity of these effects is directly related to the type and dose of radiation and dose-response relation. Muscle regeneration is a crucial process responsible for maintaining the integrity of muscle mass and muscle function throughout life, especially after muscle injury.^6^ One of the most likely mechanisms contributing to radiation-induced muscle damage is the inability of muscles to regenerate.^7^ Impaired muscle regeneration following irradiation may be due to an insufficient number of activated satellite cells, which are necessary for the fusion and repair of damaged muscle fibres. In addition, insufficient regeneration may be due to impaired cytokine signalling and ultimately impaired differentiation.^8^

This indicates that skeletal muscle is sensitive to ionizing radiation, especially during development. Therefore, radiotherapy in childhood can lead to muscle atrophy due to the large number of radiosensitive stem cells during a child’s growth phase.^9^

Thus, maintaining muscle mass during radiotherapy is essential for preserving patients’ functional capacity and general quality of life. In addition, radiotherapy often leads to muscle atrophy caused by inflammation, oxidative stress, satellite cell depletion and metabolic dysfunction. These effects contribute to fatigue, reduced strength and impaired recovery. Beyond its role in movement and metabolism, skeletal muscle also contributes significantly to immune regulation and the maintenance of systemic homeostasis.^10^

In this review, we described the mechanisms of skeletal muscle regeneration and how irradiation disrupts these processes, ultimately leading to muscle dysfunction and impaired tissue integrity. In addition, we have discussed the radioprotective role of melatonin, which is known for its powerful antioxidant and anti-inflammatory properties. Its ability to protect skeletal muscle during or after radiotherapy has gained increasing attention due to its effectiveness in attenuating radiation-induced muscle damage.

To date, only a limited number of studies have investigated the effects of radiotherapy on the skeletal muscles. Most of these studies have been conducted on animal models and have included both *in vitro* and *in vivo* approaches. Remarkably, to our knowledge only two studies have investigated these effects in human skeletal muscle cells.^5,11^

## Muscle regeneration process

Skeletal muscle is a highly structured tissue composed of numerous multinucleated cells known as myofibers, which are formed by the fusion of myogenic precursor cells. Despite the post-mitotic nature of its myofibers, skeletal muscle has a robust regenerative capacity in response to injury. This is due to the resident muscle stem cells, also known as “satellite cells” due to their unique anatomical position at the periphery of the myofibers.

The satellite cells were first identified by Mauro^12^ in 1961 and represent an important group of muscle stem cells, located between the sarcolemma and the basal lamina of the myofibers. These cells are normally in a quiescent state. Following muscle injury, or damage, satellite cells are activated from their quiescent state, proliferated and either contribute new myonuclei by fusing with existing muscle fibres to regenerate muscle tissue or return to quiescence replenish the stem cell pool for future needs.^13^ The maintenance and regulation of satellite cells is critically dependent on vascular endothelial cells, which serve as essential components of the skeletal muscle stem cell niche.^14^ This spatial relationship is essential for quiescence, activation, and self-renewal of satellite cells.

Importantly, satellite cell activation is not limited to the site of injury – they can become active, migrate, and divide from various locations along the myofiber. Notably, their density tends to be greater at the ends of muscle fibres, where longitudinal muscle growth typically occurs.^15^

**FIGURE 1.**
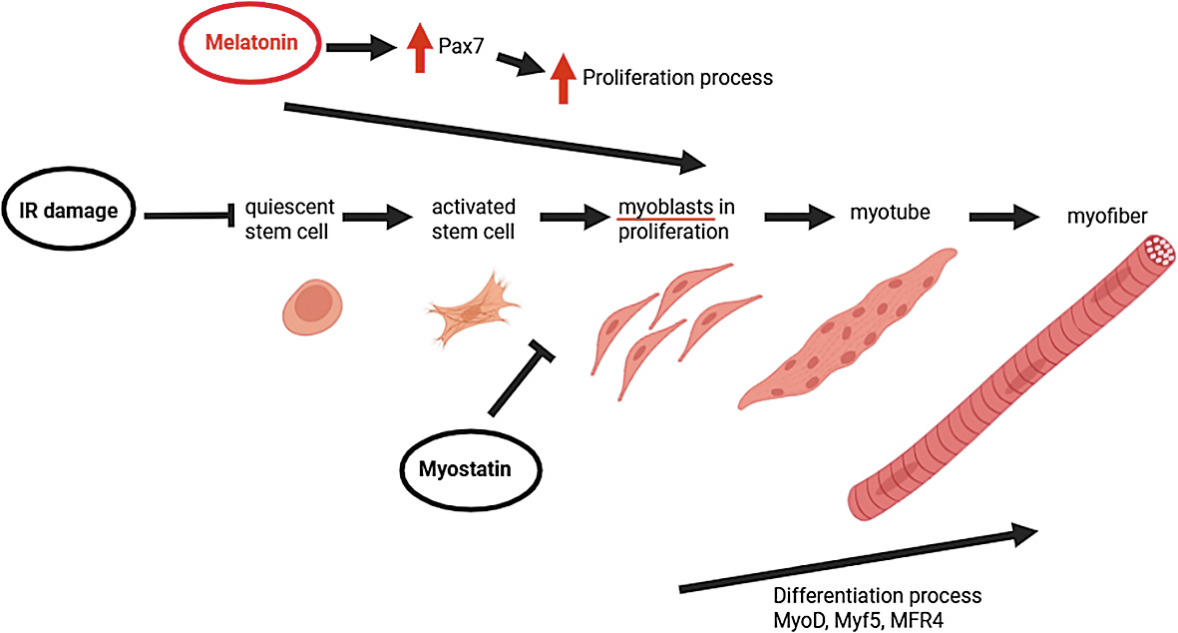
Skeletal muscle regeneration after radiotherapy. The activation and differentiation processes of the satellite cells are controlled by growth factors (with the involvement of Pax7) and myogenic regulatory factors during the regeneration of the skeletal muscle through to the formation of new muscle fibres. Melatonin induces an increase in Pax7 expression and improves skeletal muscle differentiation, while myostatin acts as a negative regulator of muscle growth and inhibits both proliferation and differentiation of myoblasts. IR = irradiation

The progression of satellite cells through the myogenic program is tightly regulated by the dynamic expression of key transcription factors. At the centre of this process is paired-box transcription factor 7 (Pax7), which is essential for the maintenance and self-renewal of satellite cells. In coordination with myogenic regulatory factors (MRFs) – including MyoD, Myf5, myogenin, and MRF4, Pax7 governs the transition of satellite cells from a quiescent state to activation, proliferation, and eventual differentiation into mature muscle fibers.^16^ The upregulation or downregulation of these factors at specific stages ensures the proper execution of muscle regeneration.^17^ When Pax7 expression remains elevated after the proliferative phase, satellite cells do not proceed to terminal differentiation. Instead, they revert to a quiescent state, a process that supports self-renewal and ensures the maintenance of the basal satellite cell pool. This regulatory mechanism is shown schematically in Figure 1 and is crucial for maintaining the longterm regenerative capacity of skeletal muscles.^18^

Alongside these, fibro-adipogenic progenitors (FAPs) – also referred to as muscle-resident mesenchymal progenitors – have emerged as key modulators of skeletal muscle homeostasis and regeneration and display multiple differentiation potential for myogenesis.^19^ Under physiological conditions, they provide pro-myogenic signals that support muscle growth, maintenance, and repair.

Following injury skeletal muscle regeneration proceeds through several phases and represents a highly coordinated process. Emerging research suggests that inflammation plays a key role between the initial injury response and effective muscle repair. Various immune cells and cytokines contribute significantly to this regeneration process.^20^

Namely, skeletal muscle injury, or myotrauma, triggers a well-orchestrated immune response that plays a pivotal role in the regeneration process. Upon injury, macrophages and other immune cells rapidly infiltrate the damaged site, initiating a sequence of molecular and cellular events. The primary functions of this immune activation are to contain and limit tissue damage, remove necrotic debris, and support the activation of regenerative pathways. This immunological response is essential for coordinating the repair process and re-establishing muscle tissue homeostasis.^20^

Macrophages, which are involved in phagocytosis of the damaged cells, move to the site of injury and release cytokines, growth factors and other substances that regulate the satellite cell. After an acute injury, macrophage infiltration peaks within 48 hours. If the macrophage response is absent, muscle regeneration does not take place. When the macrophage response is increased, there is an increase in satellite cell proliferation and differentiation.^21^ It is generally recognised that a large number of infiltrating immune cells at the site of skeletal muscle injury not only plays a crucial role in the clearance of damaged tissue, but also promotes muscle regeneration.^22,23^

Several pro-inflammatory cytokines are critically involved in the early phases of skeletal muscle regeneration following myotrauma. The most important inflammatory mediators in muscle damage include interleukin-1 (IL-1), interleukin-6 (IL-6) and tumour necrosis factor-alpha (TNF-*α*). Among these, TNF-*α* is particularly important as it is a key inflammatory mediator associated with muscle wasting in conditions such as ageing and chronic diseases. In addition, TNF-*α* plays an important role in muscle regeneration by attracting muscle stem cells to the site of injury and promoting their proliferation. This process is facilitated by the activation of the nuclear factor kappa B signalling pathway (NF-ĸB), an important transcription factor involved in the regulation of inflammation and muscle repair.^20^

Interleukin-1β (IL-1β) also plays a pivotal role in the regenerative process by promoting the recruitment of immune cells to the injury site and modulating the intrinsic properties of myoblasts (mononucleated, proliferative precursor cells derived from muscle), thereby facilitating their activation, proliferation, and differentiation.^24^

In addition, the enzyme cyclooxygenase (COX-2) plays a role in initiating the muscle repair process by regulating the inflammatory response and facilitating the activation of muscle progenitor cells. However, while COX-2 is essential for muscle regeneration in the acute phase, excessive or prolonged COX-2 activity can contribute to chronic inflammation, potentially hindering effective regeneration and promoting fibrosis.^25^

Furthermore, IL-10 important for muscle growth and regeneration, directly affects satellite cells differentiation, escorting the transition of myogenesis from the proliferative to the differentiation stage in the injured muscle.^26^

Interferon-gamma (IFN-γ) primarily recognized for its pro-inflammatory properties and its role in anti-tumour immunity, has also been shown to influence muscle regeneration.^27^ IFN-γ promotes myoblast proliferation but inhibits myogenic differentiation *in vitro*, as indicated by reduced myosin heavy chain content.^28^ This suggests that dysregulated IFN-γ expression can not only enhance the inflammatory response but also impair muscle regeneration.^29^

Collectively, these cytokines orchestrate the initial inflammatory response, which is essential for clearing damaged tissue and initiating the muscle repair cascade.^24^

In addition to the inflammatory cytokines already mentioned, it has been shown that the skeletal muscles produce and release a large number of cytokines and other signalling molecules known as myokines. These molecules play an important role in autocrine, paracrine and endocrine signalling and influence not only local muscle regeneration and inflammation, but also systemic physiological processes.^30,31^ Among them, muscle-derived interleukin-6 (IL-6), interleukin-8 (IL-8), interleukin-15 (IL-15), interleukin-1 receptor antagonist (IL-1ra), irisin, brain-derived neurotrophic factor (BDNF), secreted protein acidic and rich in cysteine (SPARC), fibroblast growth factor 21 (FGF-21) and decorin play a notable role.^8^ These myokines can act as counter-regulators to the general pro-inflammatory cytokines and help to modulate the immune response, limit excessive inflammation and support tissue regeneration following muscle injury or strain. It is important to recognized, that IL-6 has pleiotropic functions. It acts as a pro-inflammatory cytokine when secreted by immune cells under pathological conditions via the soluble IL-6 receptor (sIL-6R), while it exerts anti-inflammatory and metabolic regulatory effects when released by contracting skeletal muscle during exercise, primarily via classical signalling through the membrane-bound IL-6 receptor (mIL-6R).^32^ In contrast, muscle myostatin (also known as GDF-8, or growth differentiation factor 8) is a member of the transforming growth factorbeta (TGF-β) family, known as negative regulator of muscle growth.^33^

## Radiotherapy induced impairment of skeletal muscle regeneration

Radiation impairs both muscle regeneration and hypertrophy by damaging satellite cells. It is believed to disrupt satellite cell division by inducing DNA strand breaks. If only one strand is affected, the damage can usually be repaired by polymerases, however, if both DNA strands are broken at the same point, the damage can lead to failed cell division and cell death.^7^ However, when both DNA strands are broken at the same point, the repair mechanisms are overwhelmed. This doublestrand breakage can prevent proper cell division, leading to cellular dysfunction and, ultimately, cell death. Consequently, the impaired division of satellite cells hinders muscle regeneration and hypertrophy processes following radiation.

Low doses of radiation (2 Gy), although not harmful to the post-mitotic myonuclei in adult skeletal muscle, are sufficient to inactivate satellite cells, thereby blocking compensatory hypertrophy. After irradiation, the number of functional satellite cells appears insufficient to support muscle regeneration, either through the formation of new fibers or by fusing with existing ones.^7^

After irradiation, the development of chronic inflammation, fibrosis and vascular changes – hallmarks of late radiation effects – is associated with the activity of various regulatory factors (cytokines, myokines, myogenic regulatory factors [MRFs]) involved in skeletal muscle homeostasis and repair. A variety of cell types – such as endothelial cells, smooth muscle cells, fibro-adipogenic progenitors (FAPs), immune cells, nerve-associated cells and others – contribute to the regulation of muscle mass and tissue homeostasis.^34^ Disruption of skeletal muscle homeostasis leads to dynamic changes in the muscle microenvironment, resulting in altered cell composition and functional interactions between resident cell populations.^11,34^

Vascular endothelial cells play a vital role in maintaining the function of muscle satellite cells and regulating the infiltration of immune cells, both of which are essential for the inflammatory and regenerative responses following muscle injury.^19^

FAPs are increasingly recognized as key modulators of muscle homeostasis and regeneration, in response to acute injury and in the context of pathological muscle degeneration. During muscle injury, FAPs are crucial for orchestrating the repair process through their crosstalk with muscle stem cells and immune cells.^15^

However, when this regulatory balance is disrupted, FAPs can contribute to pathological outcomes, including fibrosis, intramuscular fat infiltration, and impaired regeneration. Recent studies have also identified distinct FAP subpopulations and secreted factors that exhibit differential responses to acute injury and chronic dysfunction, potentially influencing disease progression and regenerative outcomes.^35,36^

Collao *et al*., reported on the role of FABs in radiation-induced muscle pathology in juvenile male mice exposed to a single dose of 16 Gy of ionizing radiation. This dose was selected to model the long-term skeletal muscle fibrosis observed in cancer survivors after radiotherapy. The authors found that a dose of 16 Gy is biologically equivalent to 60 Gy delivered in 2 Gy fractions, which is a common approach in clinical cancer therapy.^37^

Their findings suggest that FAPs contribute to long-term skeletal muscle atrophy and fibrosis following juvenile radiation exposure and indicate that radiation can reduce muscle regenerative capacity and induce fibrosis, partly through adverse effects on muscle satellite cells.

In addition, radiotherapy induces significant alterations in skeletal muscle metabolism and structure, contributing to muscle degradation and impaired function.^6^

Pronounced histological alterations in irradiated skeletal muscle, including swollen and hyalinized muscle fibres, as well as the loss of normal striations, was observed many years ago by Gerstenr *et al*.^38^ They studied the effects of high-intensity X-radiation on skeletal muscle frog muscle irradiated with doses greater than 50 kilorads (kr) and from rabbit muscle irradiated with a dose of 72 kr, resulting in severe histological changes.

These changes are often accompanied by a significant infiltration of polymorphonuclear leukocytes, indicating a strong inflammatory response and exacerbating the structural degradation and compromised integrity of muscle tissue following exposure to ionizing radiation. In addition, alteration of glycogen production was observed.^39^

Additional studies in rats confirmed the association between radiotherapy with single doses of 20–30 Gy with alterations in muscle morphology, fibrosis and muscle fibre atrophy, whilefractionated doses of more than 14 Gy (two fractionated doses of 15 Gy each, totalling 30 Gy) has been shown to induce irreversible endothelial apoptosis and impair vascular integrity.^40–42^ This vascular damage contributes to increased muscle fibrosis, changes in fibre morphology and impaired muscle contractility^43^, ultimately leading to long-term deficits in muscle regeneration and tissue perfusion.^44^

Two important muscle structural proteins, titin and nebulin are essential for maintaining the highly organized architecture of skeletal muscle by precisely regulating the assembly and stabilization of myosin and actin filaments. Following low doses of irradiation, degradation of these proteins has been observed in rabbit skeletal muscle fibres, leading to a loss of structural integrity and a reduction in the elastic properties of muscle tissue.^45^

The increased availability of amino acids, especially alanine and glutamine, observed after 15 Gy of gamma irradiation in mice is primarily attributed to the increased protein degradation in the skeletal muscles that occurs immediately after irradiation. This radiation-induced disruption of protein metabolism could play an important pathogenic role in the development of radiation-induced myopathy.^46^

Further changes were observed in the membrane of the sarcoplasmic reticulum after irradiation of rabbits (X-ray irradiation with a dose of 0.21 C/kg, which corresponds to about 1.5 Gy), which are mainly due to enzymatic changes in Ca^2+^-ATPase activity. These results show that ionizing radiation can induce structural changes in the Ca2+-ATPase. Conformational changes in enzyme activity contribute to structural disruption of the sarcoplasmic reticulum membrane, which may affect calcium homeostasis and muscle contractility.^47^

Human study on muscle-based breast reconstruction have shown that the skeletal muscles used in such procedures are affected by irradiation.^11^ In this study 41 patients were treated with an average total dose of 50 Gy of fractionated radiotherapy within 6 months of breast reconstruction. The radiotherapy was administered in several fractions, as is usual in clinical practise for adjuvant radiotherapy of the breast. Significant alterations in muscle architecture, including a reduction in the number of myofibers, were observed in irradiated rectus abdominis muscle tissue. The authors concluded that ionizing radiation induces aberrant expression of catabolic, anti-myogenic (anti-myodifferentiative and antimyoproliferative) and pro-inflammatory proteins that contribute to impaired muscle regeneration and structural deterioration.

In context of myokine regulation, an *in vitro* study on human skeletal muscle myoblasts investigated the effects of irradiation on IL-6 expression and showed that these cells are very sensitive to radiation, particularly with regard to their proliferative capacity and cytokine secretion.^5^ After irradiation, an acute decrease in IL-6 secretion was observed, which was more pronounced at lower radiation doses (2-6 Gy) than at higher doses (8 Gy).^5^ This unexpected dose-response pattern suggests that the smaller decrease in IL-6 at higher doses may be due to passive IL-6 release by damaged or dying cells. All irradiated groups exhibited reduced proliferation and increased cell death compared to controls. Given that myoblast proliferation and differentiation are crucial for muscle regeneration, these effects are significant, particularly in clinical scenarios involving radiotherapy.

## Melatonin as a therapeutic agent for the skeletal muscle protection

Ionizing radiation generates reactive oxygen species (ROS) and reactive nitrogen species (RNS) that cause oxidative stress in tissues, including skeletal muscle.

Numerous studies have reported that the hormone melatonin, which is secreted by the pineal gland, has antioxidant and anti-inflammatory properties in damaged or diseased skeletal muscle.^48–51^ Melatonin is a powerful free radical scavenger that directly neutralizes ROS and RNS. This helps to reduce oxidative damage to muscle cells, including DNA, lipids and proteins, thereby protecting muscle integrity.^52,53^ In addition, melatonin increases the activity of antioxidant enzymes such as superoxide dismutase (SOD), catalase (CAT) and glutathione peroxidase (GP), which in turn helps to mitigate oxidative damage in muscle cells. Melatonin suppress the radiotherapy-induced inflammatory cytokines TNF-*α*, IL-6 and IL-1β, thereby reducing muscle inflammation and the associated catabolic processes that lead to muscle wasting. ^54^

In particular, melatonin promotes cellular differentiation. In models of muscle atrophy, upregulation of Pax7 appears to induce the proliferation of satellite cells, which subsequently form myocytes essential for future muscle regeneration.^55^ *In vitro* studies have shown that melatonin increases the expression of Pax7, thereby improving the biomechanical properties of skeletal muscle and facilitating differentiation as it shown in Figure 1.^55,56^

In addition, an inverse relationship between urinary melatonin levels and sarcopenia was observed in postmenopausal women, suggesting a protective role of melatonin against age-related muscle degeneration.^57^ Taken together, these results emphasize the potential of the melatonin/Pax7 axis as a promising therapeutic target to improve muscle healing and regeneration after injury.^56^

## Conclusions

Radiotherapy can cause both immediate and longterm damage to the skeletal muscles. The early effects are often characterized by acute damage and inflammation, while the late effects are mainly associated with fibrosis, muscle atrophy and reduced repair capacity, which can lead to chronic disability and loss of function. The severity of these effects depends on the dose and duration of radiation exposure.

Skeletal muscle regeneration is a tightly regulated process that improves muscle differentiation and promotes myogenesis.^58^ This regeneration is primarily controlled by inflammatory response and various structural proteins such as transcriptional and myogenic factors that are expressed in satellite cells and play a key role in muscle repair.

Given the crucial role of skeletal muscle myokines in muscle regeneration, special attention is required in future studies as only a limited number of experiments have been performed in the context of radiotherapy.

Furthermore, radioprotective agents are also important for promoting muscle regeneration by supporting the activation and differentiation of satellite cells, which are essential for muscle repair. Among these agents, melatonin is characterized by its multiple biological functions. It not only reduces oxidative stress and inflammation, but also promotes muscle regeneration by upregulating Pax7, an important transcription factor involved in satellite cell proliferation and muscle regeneration. By upregulating Pax7, melatonin contributes significantly to the repair and functional recovery of skeletal muscle after radiation-induced damage and is therefore an important topic for further investigation.

## References

[1] Barnett GC, West CM, Dunning AM, Elliott RM, Coles CE, Pharoah PD (2009). Normal tissue reactions to radiotherapy: towards tailoring treatment dose by genotype. Nat Rev Cancer.

[2] Baskar R. (2010). Emerging role of radiation induced bystander effects: cell communications and carcinogenesis. Genome Integr.

[3] Baskar R, Lee KA, Yeo R, Yeoh KW. (2012). Cancer and radiation therapy: current advances and future directions. Int J Med Sci.

[4] Emami B, Lyman J, Brown A, Coia L, Goitein M, Munzenrider JE (1991). Tolerance of normal tissue to therapeutic irradiation. Int J Radiat Oncol Biol Phys.

[5] Jurdana M, Cemazar M, Pegan K, Mars T. (2013). Effect of ionizing radiation on human skeletal muscle precursor cells. Radiol Oncol.

[6] Viana W, Lambertz D, Borges E, Melo J, Lambertz K, Amaral A. (2015). Late effects of radiation on skeletal muscle: an open field of research. J Biomed Sci Eng.

[7] Jurdana M. (2008). Radiation effects on skeletal muscle. Radiol Oncol.

[8] Waldemer-Streyer RJ, Kim D, Chen J. (2022). Muscle cell-derived cytokines in skeletal muscle regeneration. FEBS J.

[9] Jurdana M. (2009). Cancer cachexia-anorexia syndrome and skeletal muscle wasting. Radiol Oncol.

[10] Rogeri PS, Gasparini SO, Martins GL, Costa LKF, Araujo CC, Lugaresi R (2020). Crosstalk between skeletal muscle and immune system: which roles do IL-6 and glutamine play?. Front Physiol.

[11] Tran NV, Evans GR, Kroll SS, Baldwin BJ, Miller MJ, Reece GP (2000). Postoperative adjuvant irradiation: effects on transverse rectus abdominis muscle flap breast reconstruction. Plast Reconstr Surg.

[12] Mauro A. (1961). Satellite cell of skeletal muscle fibers. J Biophys Biochem Cytol.

[13] Relaix F, Bencze M, Borok MJ, Der Vartanian A, Gattazzo F, Mader R (2021). Perspectives on skeletal muscle stem cells. Nat Commun.

[14] Verma M, Asakura Y, Murakonda BSR, Pengo T, Latroche C, Chazaud B (2018). Muscle satellite cell cross-talk with a vascular niche maintains quiescence via VEGF and Notch signaling. Cell Stem Cell.

[15] Yin H, Price F, Rudnicki MA. (2013). Satellite cells and the muscle stem cell niche. Physiol Rev.

[16] Zammit PS. (2017). Function of the myogenic regulatory factors Myf5, MyoD, Myogenin and MRF4 in skeletal muscle, satellite cells and regenerative myogenesis. Semin Cell Dev Biol.

[17] Seale P, Sabourin LA, Girgis-Gabardo A, Mansouri A, Gruss P, Rudnicki MA (2000). Pax7 is required for the specification of myogenic satellite cells. Cell.

[18] Olguin HC, Yang Z, Tapscott SJ, Olwin BB. (2007). Reciprocal inhibition between Pax7 and muscle regulatory factors modulates myogenic cell fate determination. J Cell Biol.

[19] Chen W, You W, Valencak TG, Shan T. (2022). Bidirectional roles of skeletal muscle fibro-adipogenic progenitors in homeostasis and disease. Ageing Res Rev.

[20] Yang W, Hu P. (2018). Skeletal muscle regeneration is modulated by inflammation. J Orthop Translat.

[21] Vierck J, O’Reilly B, Hossner K, Antonio J, Byrne K, Bucci L (2000). Satellite cell regulation following myotrauma caused by resistance exercise. Cell Biol Int.

[22] Tidball JG. (2017). Regulation of muscle growth and regeneration by the immune system. Nat Rev Immunol.

[23] Tidball JG, Villalta SA. (2010). Regulatory interactions between muscle and the immune system during muscle regeneration. Am J Physiol Regul Integr Comp Physiol.

[24] Alvarez AM, DeOcesano-Pereira C, Teixeira C, Moreira V. (2020). IL-1β and TNF-α modulation of proliferated and committed myoblasts: IL-6 and COX-2-derived prostaglandins as key actors in the mechanisms involved. Cells.

[25] Wang L, Wang M, Tang X, Zhang M, Zhang K, Gao B. (2024). Mechanistic studies of cyclooxygenase-2 (COX-2) in skeletal muscle cells during rotator cuff injury: an in vitro study. Physiol Res.

[26] Fabbrizio P, Margotta C, D’Agostino J, Suanno G, Quetti L, Bendotti C (2023). Intramuscular IL-10 administration enhances the activity of myogenic precursor cells and improves motor function in ALS mouse model. Cells.

[27] Babaeijandaghi F, Paiero A, Long R, Tung LW, Smith SP, Cheng R (2022). TNFα and IFNγ cooperate for efficient pro- to anti-inflammatory transition of macrophages during muscle regeneration. Proc Natl Acad Sci U S A.

[28] Grzelkowska-Kowalczyk K, Wicik Z, Majewska A, Tokarska J, Grabiec K, Kozłowski M (2015). Transcriptional regulation of important cellular processes in skeletal myogenesis through interferon-γ. J Interferon Cytokine Res.

[29] Zhuang S, Russell A, Guo Y, Xu Y, Xiao W. (2023). IFN-γ blockade after genetic inhibition of PD-1 aggravates skeletal muscle damage and impairs skeletal muscle regeneration. Cell Mol Biol Lett.

[30] Pedersen BK, Febbraio MA. (2012). Muscles, exercise and obesity: skeletal muscle as a secretory organ. Nat Rev Endocrinol.

[31] Whitham M, Febbraio MA. (2016). The ever-expanding myokinome: discovery challenges and therapeutic implications. Nat Rev Drug Discov.

[32] Rose-John S. (2012). IL-6 trans-signaling via the soluble IL-6 receptor: importance for the pro-inflammatory activities of IL-6. Int J Biol Sci.

[33] Carnac G, Vernus B, Bonnieu A. (2007). Myostatin in the pathophysiology of skeletal muscle. Curr Genomics.

[34] Theret M, Rossi FMV, Contreras O. (2021). Evolving roles of muscle-resident fibroadipogenic progenitors in health, regeneration, neuromuscular disorders, and aging. Front Physiol.

[35] Uezumi A, Fukada S, Yamamoto N, Takeda S, Tsuchida K. (2010). Mesenchymal progenitors distinct from satellite cells contribute to ectopic fat cell formation in skeletal muscle. Nat Cell Biol.

[36] Joe AWB, Yi L, Natarajan A, Le Grand F, So L, Wang J (2010). Muscle injury activates resident fibro/adipogenic progenitors that facilitate myogenesis. Nat Cell Biol.

[37] Collao N, D’Souza D, Messeiller L, Pilon E, Lloyd J, Larkin J (2023). Radiation induces long-term muscle fibrosis and promotes a fibrotic phenotype in fibro-adipogenic progenitors. J Cachexia, Sarcopenia Muscle.

[38] Gerstner HB, Lewis RB, Richey EO. (1953). Early effects of high intensity X-radiation on skeletal muscle. J Gen Physiol.

[39] Ahlersová E, Ahlers I, Slavkovská E, Praslicka M. (1981). Metabolic changes after non-lethal X-irradiation of rats. I. Carbohydrates, hormones. Folia Biol (Praha).

[40] Pitkanen MA, Hopewell JW. (1983). Functional changes in the vascularity of the irradiated rat femur. Implications for late effects. Acta Radiol Oncol.

[41] Adams GR, Caiozzo VJ, Haddad F> Baldwin KM. (2002). Cellular and molecular responses to increased skeletal muscle loading after irradiation. Am J Physiol Cell Physiol.

[42] Garcia-Barros M, Paris F, Cordon-Cardo C, Lyden D, Rafii S, Haimovitz-Friedman A (2003). Tumor response to radiotherapy regulated by endothelial cell apoptosis. Science.

[43] Avelino SOM, Neves RM, Sobral-Silva LA, Tango RN, Federico CA, Vegian MRC (2023). Evaluation of the effects of radiation therapy on muscle contractibility and skin healing: an experimental study of the cancer treatment implications. Life (Basel).

[44] Gallet P, Phulpin B, Merlin JL, Leroux A, Bravetti P, Mecellem H (2011). Longterm alterations of cytokines and growth factors expression in irradiated tissues and relation with histological severity scoring. PLoS One.

[45] Horowits R, Kempner ES, Bisher ME, Podolsky RJ. (1986). A physiological role for titin and nebulin in skeletal muscle. Nature.

[46] Schwenen M, Altman KI, Schroder W. (1989). Radiation-induced increase in the release of amino acids by isolated, perfused skeletal muscle. Int J Radiat Biol.

[47] Khizhniak SV, Voitsitskii VM, Ostapchenko SG, Kucherenko NE. (1990). [The effect of ionizing radiation on Ca 2+-ATPase activity from the sarcoplasmic reticulum of rabbit skeletal muscles]. [Rusian]. Ukr Biokhim Zh (1978).

[48] Wang Y, Guo X, Wang L, Yu H. (2023). Melatonin improves muscle injury and differentiation by increasing Pax7 expression. Int J Mol Sci.

[49] Jang YC, Van Remmen H. (2012). Melatonin restores muscle regeneration and enhances muscle function after crush injury in rats. J Pineal Res.

[50] Ge X, Wang C, Yang G, Maimaiti D, Hou M, Liu H (2024). Enhancement of mitochondrial energy metabolism by melatonin promotes vascularized skeletal muscle regeneration in a volumetric muscle loss model. Free Radic Biol Med.

[51] Zhu GZ, Zhao K, Li HZ, Wu DZ, Chen YB, Han D (2024). Melatonin ameliorates age-related sarcopenia by inhibiting fibrogenic conversion of satellite cells. Mol Med.

[52] Mihandoost E, Shirazi A, Mahdavi SR, Aliasgharzadeh A. (2014). Can melatonin help us in radiation oncology treatments?. Biomed Res Int.

[53] Tan DX, Manchester LC, Esteban-Zubero E, Zhou Z, Reiter RJ. (2015). Melatonin as a potent and inducible endogenous antioxidant: synthesis and metabolism. Molecules.

[54] Najafi M, Shirazi A, Motevaseli E, Geraily G, Norouzi F> Heidari M (2017). The melatonin immunomodulatory actions in radiotherapy. Biophys Rev.

[55] Penna F, Costamagna D, Fanzani A, Bonelli G, Baccino FM, Costelli P. (2010). Muscle wasting and impaired myogenesis in tumor-bearing mice are prevented by ERK inhibition. PLoS One.

[56] Su CM, Tsai CH, Chen HT, Wu YS, Chang JW, Yang SF (2023). Melatonin improves muscle injury and differentiation by increasing Pax7 expression. Int J Biol Sci.

[57] Lee JY, Kim JH, Lee DC. (2014). Urine melatonin levels are inversely associated with sarcopenia in postmenopausal women. Menopause.

[58] Yu D, Cai Z, Li D, Zhang Y, He M, Yang Y (2021). Myogenic differentiation of stem cells for skeletal muscle regeneration. Stem Cells Int.

